# Prognostic Comparison between Oncotype DX^®^ and a 23-Gene Classifier, RecurIndex^®^, on the Taiwan Breast Cancer Population

**DOI:** 10.3390/diagnostics12112850

**Published:** 2022-11-17

**Authors:** Chuan-Hsun Chang, Po-Sheng Yang, Chia-Ming Hsieh, Ting-Hao Chen, Skye Hung-Chun Cheng, Cheng-En Yang, Chiun-Sheng Huang

**Affiliations:** 1 Department of Surgery, Cheng-Hsin General Hospital, Taipei 112201, Taiwan; 2 Department of Medicine, MacKay Medical College, New Taipei 252450, Taiwan; 3 Department of General Surgery, MacKay Memorial Hospital, Taipei 104490, Taiwan; 4 Department of Surgery, Taiwan Adventist Hospital, Taipei 105520, Taiwan; 5 Department of Medical Operation, Amwise Diagnostics Pte. Ltd., Singapore 069547, Singapore; 6 Institute of Epidemiology and Preventive Medicine, College of Public Health, National Taiwan University, Taipei 106170, Taiwan; 7 Department of Radiation Oncology, Koo Foundation Sun Yat-Sen Cancer Center, Taipei 112590, Taiwan; 8 Taitung Cancer Center, Taitung Christian Hospital, Taitung City 950405, Taiwan; 9 Department of Surgery, National Taiwan University Hospital, Taipei 300600, Taiwan; 10 College of Medicine, National Taiwan University, Taipei 100233, Taiwan

**Keywords:** breast cancer, recurrence/relapse prognosis, genomic classifier, gene expression profiling tests

## Abstract

The applicability of the Oncotype DX^®^ (Genomic Health, Inc., Redwood City, CA, USA) recurrence score (RS) in Asian populations is unclear. A 23-gene classifier, RecurIndex^®^ (Amwise Diagnostics, Pte. Ltd., Singapore), has been developed based on the gene expression profiles of early-stage breast cancer patients of ethnic Han Chinese population in Taiwan. This study aimed to compare the performance of the Oncotype DX^®^ RS with the RecurIndex^®^ recurrence index (RI) for predicting relapse-free survival. Therefore, we calculated both the RI and RS for 110 early stage breast cancer patients, with the cut-off value for high-risk recurrence set at 26 and 29 for the RS and the RI, respectively. With relapse-free interval (RFI) as the primary endpoint, the concordance between RS and RI was 78.2% (Kappa value = 0.297). For a median follow-up interval of 27 months, there was a statistically significant difference in RFI between the high- and low-risk groups defined by the RI (*p* = 0.04) but not between risk groups defined by the RS (*p* = 0.66). In conclusion, whereas there was high concordance between the RecurIndex^®^ RI and the Oncotype DX RS, the current data showed that the RI had a better discrimination for recurrence risk than the RS. Subsequent studies with larger sample sizes will be needed to confirm the superiority of the RI over the RS in the Asian population.

## 1. Introduction

Breast cancer is the most common female cancer worldwide, with an estimated 2.3 million cases of newly diagnosed patients in 2020 [1]. Among node- and hormone receptor (HR)-negative patients, surgery coupled with adjuvant chemotherapy can effectively reduce the risk of recurrence [2]. In contrast, because of the relatively low recurrence rate in HR-positive patients, it can be difficult to determine whether adjuvant chemotherapy is indeed necessary [3,4]. The TAILORx trial of the Oncotype DX^®^ test has provided robust evidence that patients with HR-positive, human epidermal growth factor receptor 2 (HER2)-negative, and node-negative breast cancer can be spared from adjuvant chemotherapy without compromising the final clinical recurrence outcomes [5].

Genomic tests for breast cancer prognosis, such as Oncotype DX^®^, EndoPredict^®^ (Myriad Genetics, Inc., Salt Lake City, UT, USA), and MammaPrint^®^ (Agendia, Amsterdam, The Netherlands), can help guide treatment decisions. However, these commercially available assays have been developed based on the genomics of Western populations [6]. The incidences and prognoses of the early stage breast cancer patients can vary significantly by different ethnicities [7]. For example, Yeo et al. reported that breast tumor characteristics in young Asian women are different from those of their Western counterparts, which highlights the need for comprehensive genomic data on Asian populations to support the decision-making during treatment of breast cancer [8]. In contrast with the non-Hispanic white population, the pattern of breast cancer-specific mortality among Asian node-negative patients is different from that in non-Hispanic white reference populations [9]. Hence, a 23-gene classifier was developed using gene expression profiles from the breast cancer patients in Taiwan [10,11].

The 23-gene classifier, RecurIndex^®^, was initially developed using the microarray platform technology [10,11,12] to evaluate the prognosis capability of the test in classifying Asian breast cancer patients into two categories, high and low risks of distant recurrence (DR). This 23-gene genomic test comprises 20 breast cancer recurrence-related genes with three housekeeping genes and six clinical factors (the age at first diagnosis, the nodal stage, the tumor grading, the tumor size, the estrogen receptor (ER) status, and the lymphovascular invasion (LVI) status) [12]. In a previous study, we adopted the NanoString nCounter system for performing the 23-gene classifier test and compared its risk partitioning capability with the Oncotype DX^®^ [13]. However, for considerations on cost, the reverse-transcriptase (RT) quantitative polymerase chain reaction (qPCR) platform technology was later adopted over the nCounter and the microarray platform systems. In the present study, we assessed the performance of Oncotype DX^®^ in the Taiwanese population and compared the prognostic performance of the test with the 23-gene classifier using the same patients’ data obtained from the RT-PCR platform. 

## 2. Methods and Materials

### 2.1. Study Population

**Figure 1:**
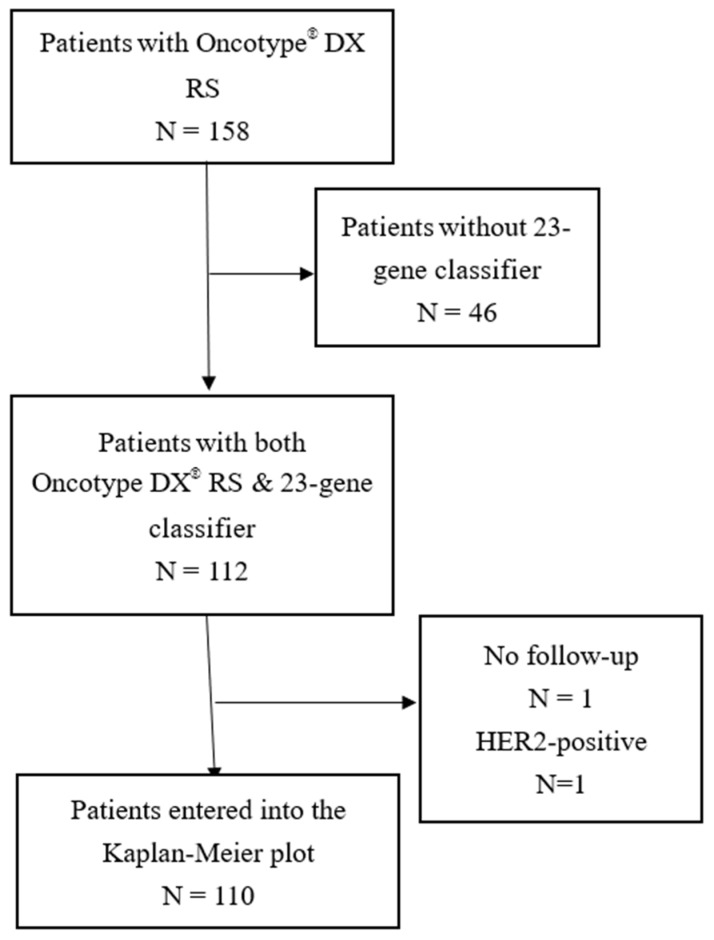
Patient selection.

Early stage breast cancer patients, who had Oncotype DX^®^ test results from three medical centers in Taiwan, were recruited to participate in this study and had their formalin-fixed, paraffin-embedded (FFPE) tumor tissue sections tested with the 23-gene classifier. A total of 110 patients with the following inclusion criteria were included in this study (Figure 1): (i) Female subject aged 20 years old or older; (ii) subject with signed informed consent form; (iii) subject with adequate FFPE tumor tissue samples (iv) subject with mastectomy or breast-conserving surgery (BSC) as the first-line treatment; (v) subject with pT1-3 disease; (vi) subject with pN0-1 disease; (vii) subject with ER or PR immunohistochemistry (IHC) positive- or negative-primary breast cancer, receiving or having received adjuvant hormonal therapy; (viii) subject with HER2 IHC positive-, equivocal-, or negative-primary breast cancer; (ix) subject with known LVI status; and (x) subject with tumor grade information. Patients were excluded if they met one or both of the following criteria: (i) subject that received pre-operative (i.e., neoadjuvant) chemotherapy or radiotherapy; (ii) subject receiving BCS but receiving no adjuvant radiotherapy for treating breast cancer; and (iii) subject was not Taiwanese based on the electronic medical records. All the clinical factors and the information on follow-up were obtained from the electronic medical records. The clinical and histopathological factors such as the number of positive lymph nodes, the tumor grading (I, II, and III), the tumor size (cm), the ER status (positive/negative), and the LVI status (yes/no) were reviewed by an independent pathologist to determine them. In addition, to calculate the RurcuIndex score, the tumor size was transformed into tumor stage (T1: <=2 cm, T2: >2 cm to <=5 cm, T3: >5 cm) and the number of positive nodes was transformed into nodal stage (N0: no positive node, N1: 1–3 positive node, and N2: 4–9 positive nodes). The institutional review board (IRB) of each participating medical center approved the protocol of this study (IRB numbers: MacKay Memorial Hospital, 17CT040be; Taiwan Adventist Hospital, 107-E-05; and Cheng Hsin General Hospital, 108B-09). 

### 2.2. RecurIndex, 23-Gene Classifier

The development of the 23-gene classifier has been described previously [10,11,12]. The primer pairs of the 23-genes were set into the 96-well plates to conduct RT-PCR by using the total RNA isolated from the FFPE tumor tissues. RNA was extracted from the FFPE tissues with the RNeasy FFPE Kit (Qiagen, Valencia, CA, USA). In addition, the RT reaction was conducted at 42 °C for 15 min and the PCR procedure was performed on the ABI7500fast instrument (Thermo Fisher, Waltham, MA, USA) under the standard mode with 40 cycles at 95 °C 15 s and 60 °C for 45 s.

### 2.3. Determination of Cut-Off Values

For the risk determination based on data from the RT-PCR platform, the cut-off value for the 23-gene classifier was set to 29, using the receiver operating characteristic (ROC) analysis to determine the best combination of sensitivity and specificity. Patients with the 23-gene classifier recurrence index (RI) ≥ 29 were defined as having high-risk of recurrence, and those with 23-gene classifier RI < 29 were defined as low risk. For the Oncotype DX^®^ RS, we defined the risk groups as in the TAILORx trial, with a recurrence score (RS) < 25 indicating non-high-risk, and scores ≥ 25 indicating high-risk. 

### 2.4. Clinical Performance and Concordance between Oncotype DX^®^ and the 23-Gene Classifier

The clinical performance of the two assays was evaluated by calculating the negative predictive value (NPV), positive predictive value (PPV), sensitivity, and specificity of the 23-gene classifier and the Oncotype DX^®^ RS for predicting the actual status of recurrence in each patient within five years after surgery. In addition, a Kaplan–Meier plot was used to observe the survival time, with the log-rank test used to compare the relapse-free interval (RFI) between the high- and low-risk groups as determined by the two assays. Furthermore, we calculated Cohen’s kappa coefficient to evaluate the agreement between the Oncotype DX^®^ RS and the 23-gene classifier RI.

## 3. Results

### 3.1. Characteristics of the Study Population

Table 1 shows the baseline characteristics of the recruited patients. Among the 110 patients eligible for this study, the mean (SD) age was 51.79 (11.05), and the median follow-up time was 26.98 months (interquartile range 14.16–45.21). Most of the patients included in this study were N0 (76.58% and N1 23.42%, respectively), with T1 or T2 disease (57.27% and 41.82%, respectively), and N0 or N1 disease (76.36% and 23.64%, respectively). Twenty patients had grade I tumors (18.18%), 82 grade II (74.55%), and the remaining 8 patients grade III (7.27%). Two patients had recurrence within the follow-up period.

### 3.2. Patient Characteristics as Determined by the 23-Gene Classifier 

Table 2 shows the characteristics of patients in the high- and low-risk groups as defined by the 23-gene classifier. The high-risk patients had the lower age (49.71 (11.96)) than low-risk patients (52.37 (10.79)) and recurrence (8.33% in the high-risk group vs. 0.00% in the low-risk group, *p* = 0.046). The distribution of tumor grade was nearly statistically significantly between the low- and high-risk groups of patients (*p* = 0.068).

### 3.3. Patient Characteristics as Determined by the Oncotype DX^®^

Table 3 shows the characteristics of patients in the high- and low-risk groups as defined by the Oncotype DX^®^. Regarding the tumor grade, there were significantly more grade III in the high-risk group (25.00%) than in the low-risk group (4.26%, *p* = 0.012). In addition, among the only two patients that had recurrence, one was in the high-risk whereas one was in the low-risk group.

### 3.4. Clinical Performance of the 23-Gene Classifier RI and Oncotype DX^®^ RS

In Table 4, the 23-gene classifier RI shows a good prognostic capability for the early breast cancer patients, as the two (100%) recurrence cases were classified into the high-risk group (with a PPV of 8.3%), while no recurrence cases were classified in the low-risk group (with an NPV of 100%).

Table 5 shows the prognostic capability of Oncotype DX^®^ RS for early breast cancer patients. Only one (66.67%) of the two recurrence cases were classified into the high-risk group (with a PPV of 5.6%), whereas one recurrence case was classified into the low-risk group (with an NPV of 98.9%).

**Figure 2:**
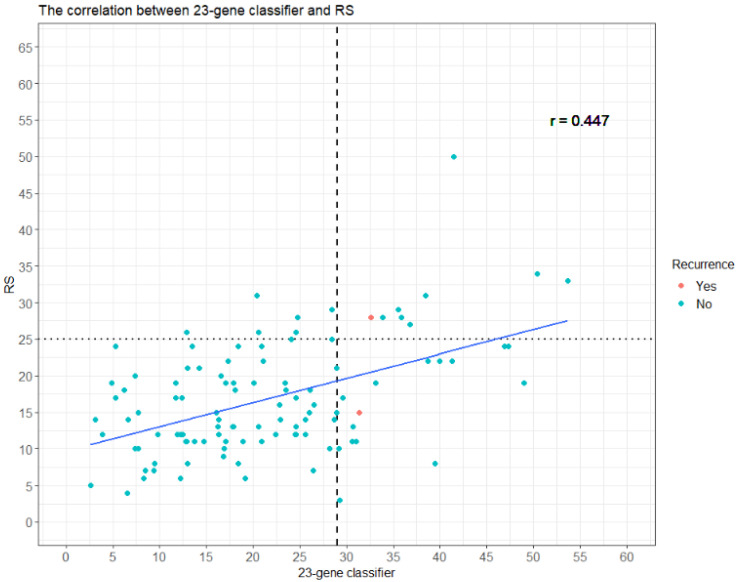
Scatter plot for 23-gene classifier RI and Oncotype DX^®^ RS.

Table 6 shows the concordance between the Oncotype DX^®^ RS and the 23-gene classifier RI. Among the 110 patients, 24 (22%) and 86 (78%) were categorized by the 23-gene classifier RI as high- and low-risk, respectively, whereas 16 (15%) and 94 (85%) were classified by the Oncotype DX^®^ RS as high- and non-high-risk, respectively. For a total of 88 patients (80%), the two assays agreed: 9 (8.2%) into the high-risk and 79 (72%) into the low/non-high-risk groups as determined by both the Oncotype DX^®^ RS and the 23-gene classifier RI. For concordance between the 23-gene classifier RI and the Oncotype DX^®^ RS, the Cohen’s kappa coefficient was 0.297, indicating a low concordance between the two assays. As shown in Figure 2, there was a significant correlation between the 23-gene classifier RI and the Oncotype DX^®^ RS (*r* = 0.447, *p* < 0.001).

**Figure 3:**
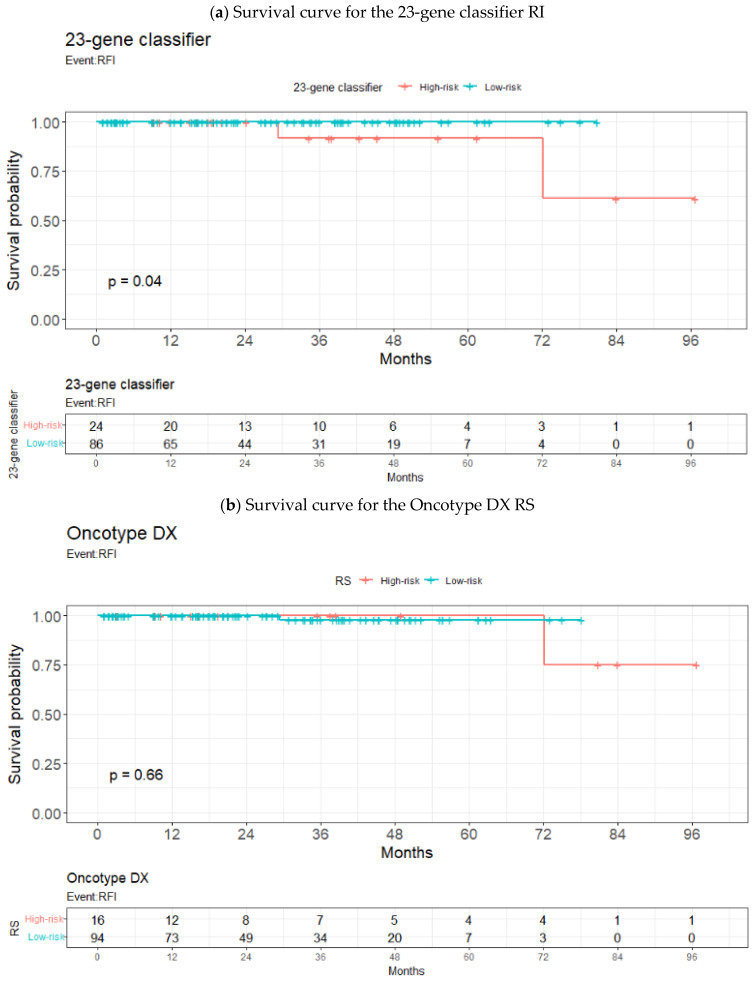
Kaplan–Meier plot for high-risk and low-risk/non-high-risk patients based on (**a**) the 23-gene classifier RI and (**b**) Oncotype DX^®^ RS.

In Figure 3, the Kaplan–Meier plot shows a significant difference between the high- and low-risk groups as partitioned by the 23-gene classifier RI (*p* = 0.04). The 7-year RFI for the low-risk group was 100%, whereas that for the high-risk group was 61% (interquartile range 27–100%). In comparison, for the Oncotype DX^®^ RS, the 7-year RFI for the low-risk group was 98% (93–100%) and that for the high-risk group was 75% (43–100%) (*p* = 0.66). 

## 4. Discussion

It has been shown that most of the early stage breast cancer patients of the luminal subtype remained non-recurrent even when the patients were not treated with adjuvant chemotherapy [3]. It would thus be beneficial if more prognostic information could be obtained by testing with tumor tissue specimens of the patients. For example, if a patient is classified as low-risk for future recurrence by a gene-expression profiling assay, the adjuvant chemotherapy may possibly be omitted from the treatment regimens. In fact, the TAILORx trial of the Oncotype DX^®^ assay has shown that patients with breast cancer of the luminal subtype (i.e., HR-positive and HER2-negative) and with node-negative status can be spared from adjuvant chemotherapy [5]. 

However, the Oncotype DX^®^ assay and other breast cancer prognostic assays on the market were developed based on the Western populations and may not be suitable for the Asian population [9]. In fact, breast cancer tumor characteristics of Asian women have been shown to differ from those of Western populations. For example, Hoskins et al. showed that the hazard ratio (HR) of recurrence in Asians/Pacific Islanders was 0.67 (confidence interval 0.50–0.90) when compared to non-Hispanic white women with node-negative cancer, after adjusting for confounding factors such as age, year of diagnosis, tumor size, progesterone receptor status, type of surgery, and administration of radiotherapy and chemotherapy [9]. More specifically, the hazard ratio was 0.65 (confidence interval 0.42–0.99) for Asians/Pacific Islanders patients with an RS of 11-25 when compared with non-Hispanic white women populations [9], suggesting that the RS of the Oncotype DX^®^ test may overestimate recurrence risk of Asian populations. In addition, our preliminary analysis in 44 Belgium patients of GSE45255 [14] showed different partitioning between Oncotype DX^®^ and the genetic model of RecurIndex^®^. There were 35 high-risk subjects determined by Oncotype DX^®^, whereas 11 high-risk subjects were determined by RecurIndex^®^, which shows that the prognostic capability depends on the target population of the prognostic tool. The details of the results are shown in the Supplementary Table S1. It is thus important to develop an assay for the prognosis of recurrence of breast cancer in patients of Asian descent. RecurIndex^®^, a 23-gene classifier, was thus developed based on the ethnic Han Chinese breast cancer population in Taiwan to meet unmet clinical needs [10].

In the current study, we studied the performance of both the RecurIndex^®^ and the Oncotype DX^®^ assays and compared them against each other for correlation and concordance. The 23-gene classifier for recurrence prognosis of breast cancer showed good performance in predicting the outcomes of any recurrence within 5 years after surgery since all of the 86 patients classified as low-risk by the assay were free of relapse during the 5-year follow-up period, with the NPV determined to be 100% (Table 2). Taken together with the fact that the sensitivity was also determined to be 100% (Table 2), the low-risk patients may thus consider forgoing adjuvant chemotherapy to avoid the potential side effects. 

In contrast, the Oncotype DX^®^ assay had a less optimal performance with the NPV and sensitivity determined to be 98.9% and 66.7%, respectively. Still, even though the performance of the Oncotype DX^®^ assay was not as optimal as the RecurIndex^®^ assay, there was moderate correlation (*r* = 0.447, *p* < 0.001) and concordance (Cohen’s kappa = 0.297) between the two assays (Table 4), suggesting that the RecurIndex^®^ assay may have potential in providing prognostic information on breast cancer for patients other than ethnic Han Chinese. Further studies will be needed to reveal such potentials.

It should be noted that there are some limitations in this study. Firstly, most of the participants in this study had the luminal subtype of breast cancer. This subtype has a relatively good prognosis when compared to other subtypes, but it is also associated with late recurrence, i.e., recurrence in more than five years after receiving the primary surgery treatment [3]. Since the follow-up of the participants in this study was relatively short, with only 11 patients having a 5-year follow-up period, the studied cohort may thus not provide enough power to draw any solid conclusion. Secondly, due to the features of the early stage breast cancer, the number of recurrence events in this cohort was small and thus may not be able to provide a precise estimation. This made it difficult to estimate the benefits of chemotherapy through statistical methods, such as by the stratified analysis or the Cox proportional hazards regression modeling. In addition, the median follow-up was only 26.98 months, which was too short to observe enough events, especially in the luminal-type patients.

In summary, the current study showed that the 23-gene classifier, RecurIndex^®^ assay, which was developed based on the Asian population, may be used to identify low-risk breast cancer patients of Asian descendant. To further validate the performance of the 23-gene classifier, a larger cohort and a longer follow-up will be needed.

## Figures and Tables

**Table 1:** Characteristics of the study population (N = 110).

Variable	Patient Number
Age, mean (SD)	51.79 (11.05)
Nodal stage	
N0	84 (76.36%)
N1	26 (23.64%)
Grade	
I	20 (18.18%)
II	82 (74.55%)
III	8 (7.27%)
Tumor stage	
T1	63 (57.27%)
T2	46 (41.82%)
T3	1 (0.91%)
Recurrence	
Yes	2 (1.82%)
No	108 (98.18%)
Follow-up interval, median (IQR)	26.98 (14.16, 45.21)

**Table 2:** Patient characteristics according to the 23-gene classifier.

Characteristic	23-Gene Classifier ≥ 29 n = 24	23-Gene Classifier < 29 n = 86	*p*-Value
Age (SD)	49.71 (11.96)	52.37 (10.79)	0.3
Nodal stage			0.3
N0	16 (66.67%)	68 (79.07%)	
N1	8 (33.33%)	18 (20.93%)	
Grade			0.068
I	2 (8.33%)	18 (20.93%)	
II	18 (75.00%)	64 (74.42%)	
III	4 (16.67%)	4 (4.65%)	
Tumor stage			0.6
T1	12 (50.00%)	51 (59.30%)	
T2	12 (50.00%)	34 (39.53%)	
T3	0 (0.00%)	1 (1.16%)	
Relapse			0.046
Yes	2 (8.33%)	0 (0.00%)	
No	22 (91.67%)	86 (100.0%)	
Follow-up interval (IQR)	26.82 (17.68, 47.86)	26.98 (12.86, 44.30)	0.339

**Table 3:** Patient characteristics according to the Oncotype DX^®^ RS.

Characteristic	Oncotype ≥ 25, n = 16	Oncotype < 25, n = 94	*p*-Value
Age (SD)	53.12 (7.19)	51.56 (11.60)	0.5
Nodal stage			>0.9
N0	12 (75.00%)	72 (76.60%)	
N1	4 (25.00%)	22 (23.40%)	
Grade			0.012
I	1 (6.25%)	19 (20.21%)	
II	11 (68.75%)	71 (75.53%)	
III	4 (25.00%)	4 (4.26%)	
Tumor stage			0.6
T1	8 (50.00%)	55 (58.51%)	
T2	8 (50.00%)	38 (40.43%)	
T3	0 (0.00%)	1 (1.06%)	
Relapse			0.3
Yes	1 (6.25%)	1 (1.06%)	
No	15 (93.75%)	93 (98.94%)	
Follow-up interval (IQR)	28.34 [13.91, 54.78]	26.98 [14.24, 44.30]	0.593

**Table 4:** Clinical performance of the 23-gene classifier RI.

23-Gene Classifier RI	Recurrence		Total
	Yes	No	
high-risk	2 (1.8%)	22 (20%)	24 (22%)
low-risk	0 (0%)	86 (78%)	86 (78%)
Total	2 (1.8%)	108 (98%)	110 (100%)

Agreement: 0.800; Sens: 1.000; Spec: 0.796; PPV: 0.083; NPV: 1.000.

**Table 5:** Clinical performance of the Oncotype DX^®^ RS.

Oncotype Dx	Recurrence		Total
	Yes	No	
high-risk	1 (0.9%)	15 (14%)	16 (15%)
low-risk	1 (0.9%)	93 (84%)	94 (85%)
Total	2 (1.8%)	108 (98%)	110 (100%)

Agreement: 0.836; Sens: 0.500; Spec: 0.843; PPV: 0.056; NPV: 0.989.

**Table 6:** Concordance between Oncotype DX^®^ RS and the 23-gene classifier RI.

23-Gene Classifier RI	Oncotype DX^®^ RS		Total
	High-Risk	Non-High-Risk	
high-risk	9 (8.2%)	15 (14%)	24 (22%)
low-risk	7 (6.4%)	79 (72%)	86 (78%)
Total	16 (15%)	94 (85%)	110 (100%)

Agreement: 0.782; Kappa: 0.297.

## Data Availability

Supplementary data available in a publicly accessible repository. The supplementary analysis presented in this study are openly available in the GEO database (accession number(s) GSE45255). at [https://doi.org/10.1186/gb-2013-14-4-r34]. Data of the main analysis sharing not applicable No new data were created or analyzed in this study. Data sharing is not applicable to this article.

## Supplementary Materials

The following supporting information can be downloaded at: https://www.mdpi.com/article/10.3390/diagnostics12112850/s1, Table S1: The demographic of the Belgium population in GSE45255; Table S2: Confused matrix of Oncotype DX and distant metastasis; Table S3: Confused matrix of RecurIndex and distant metastasis.
