# Supplementary material for: Prognostic Comparison between Oncotype DX® and a 23-Gene Classifier, RecurIndex®, on the Taiwan Breast Cancer Population
Source: Diagnostics (Basel). 2022 Nov 17;12(11):2850. doi: 10.3390/diagnostics12112850 (PMC9689820; doi:10.3390/diagnostics12112850)
Supplement: Supplementary file 1 [file diagnostics-12-02850-s001.zip › diagnostics-1933261-supplementary.pdf]

Table S1. The demographic of the Belgium population in GSE45255**.**

Variables	n=44
**Oncotype DX**	
Mean (SD)	62.38 (35.52)
**RecurIndex**	
Mean (SD)	36.52 (0.65)
**Oncotype DX risk**	
High-risk (%)	35 (79.55%)
Low-risk (%)	9 (20.45%)
**RecurIndex risk**	
High-risk (%)	11 (25.00%)
Low-risk (%)	33 (75.00%)
**Distant metastasis**	
Yes (%)	15 (34.09%)
No (%)	29 (65.91%)

Table S2. Confused matrix of Oncotype DX and distant metastasis.

	**Clinical outcome: DR+**		
**Characteristic**	Yes	No	**Total**
**Oncotype DX (High/Low)**			
High	13	22	35
Low	2	7	9
**Total**	15	29	44

Table S3. Confused matrix of RecurIndex and distant metastasis.

	Clinical outcome: DR+		
**Characteristic**	Yes	No	**Total**
**RecurIndex (High/Low)**			
High	3	8	11
Low	12	21	33
**Total**	15	29	44
